# Evaluation of anti-biofilm and anti-virulence effect of zinc sulfate on *Staphylococcus aureus* isolates

**DOI:** 10.1038/s41598-024-75317-0

**Published:** 2024-10-28

**Authors:** Wedad M. Abdelraheem, Heba S. Kamel, Aya Nabil Gamil

**Affiliations:** 1 Medical Microbiology and Immunology department, Faculty of Medicine, Minia University, Minia, Egypt; 2 Department of Biochemistry, Faculty of Medicine, Minia University, Minia, Egypt

**Keywords:** *Staphylococcus aureus*, Zinc sulfate, Synergistic effect, Anti-virulence, Anti-biofilm, Biochemistry, Biotechnology, Microbiology, Molecular biology

## Abstract

*Staphylococcus aureus* produces a plethora of virulence factors to invade and establish infections in the host system, and biofilms are more resistant to antibiotics than planktonic cells. In this study, we aimed to investigate the anti-virulence and anti-biofilm potentials of zinc sulfate against *S. aureus* isolates. The synergistic effect of zinc sulfate in combination with antibiotics on *S. aureus* was characterized using the checkerboard method. The influence of zinc sulfate on biofilm formation and virulence factors production by *S. aureus* was experimentally assessed. RT-qPCR was used to investigate the effect of zinc sulfate on the expression of biofilm-related genes. Zinc sulfate exhibited good antibacterial activity against *S. aureus* with a MIC of 128 µg/ml against all tested isolates. Also, the findings indicate a synergistic effect of a combination of zinc sulfate and antibiotics against the tested isolates. Zinc sulfate at 256 µg/ml concentration inhibited biofilm formation for all isolates. The expression of biofilm-related genes was significantly repressed in zinc sulfate-treated bacteria compared to untreated cells. Zinc sulfate could inhibit the hemolytic ability of *S. aureus.* Moreover, zinc sulfate-treated bacteria exhibited a significant decrease in coagulase and catalase activity relative to control untreated *S. aureus*. Our results support that zinc sulfate is a potential antimicrobial and anti-virulence agent against *S. aureus* infections.

**Supplementary Information:**

The online version contains supplementary material available at 10.1038/s41598-024-75317-0.

## Introduction

*Staphylococcus aureus* (*S. aureus*) is an important human pathogen that causes a variety of clinical infections both in nosocomial and community settings. The treatment of infections caused by *S. aureus* has become a big challenge due to the emergence of multi-drug-resistant strains^1^. Biofilm formation by *S. aureus* is reported as an important cause of treatment failure and recurrent infections. Biofilm protects the organism from antimicrobials, makes antimicrobials that cannot reach the organism, and allows it to escape killing by the host immune system^2^. *Staphylococcus aureus* is a strong biofilm producer which increases the resistance of this organism to antimicrobial agents. This resistance is predominantly implemented through the diffusion barrier action of the polysaccharide matrix. Furthermore, differences in nutrient and oxygen availability within biofilm lead to differences in bacterial growth rate and metabolic activity. Bacterial metabolic rate decreases and cell division occurs at radically lower rates producing slow-growing cells known as persister cells that are highly resistant to antimicrobial agents^3^. A gene cluster known as ica operon (*icaADBC*) is an important element in the process of biofilm formation, encoding the production of polysaccharide intercellular adhesion, and fibronectin-binding proteins A and B (fnbA and fnbB), which mediates adherence of bacteria and the accumulation of multilayer biofilm^4^. The multidrug-resistant and biofilm-producing strains of *S. aureus* are increasing speedily with time, making the treatment more difficult. In this study, we focused on studying zinc sulfate as a promising alternative or synergistic agent with antibiotics for the treatment of these serious infections. Zinc is a natural mineral that we need for proper growth and development. Zinc is also used with or without vitamin C to reduce the duration and severity of symptoms of the common cold as it enhances the immune response allowing for a better clearance of the pathogens. Zinc also improves the absorption of water and electrolytes and improves the regeneration of the intestinal epithelium^5^.

Zinc sulfate is an inorganic compound with the formula ZnSO4 and is historically known as “white vitriol”. It is on the World Health Organization’s List of Essential Medicines, a list of the most important medications needed in a basic health system. The zinc in zinc sulfate enters the body if ingested, inhaled, or by skin contact and reaches the bloodstream. Once inside, zinc spreads throughout the body, binds to proteins, and enters different organs. Scientists studied long-term exposure to zinc sulfate in rats and found no harmful adverse effects^6^. We have tried zinc sulfate as an antibacterial and anti-biofilm agent against *S. aureus* clinical isolates in this study.

## Results

### Antibacterial effect of zinc sulfate against *S. aureus* clinical isolates

The antibacterial effect of zinc sulfate was tested against *S. aureus* clinical isolates by microdilution method. Importantly, zinc sulfate shows a good antibacterial effect against *S. aureus* clinical isolate with MIC of 128 µg/ml against all isolates including *S. aureus* ATCC 29213. The minimum bactericidal concentration (MBC) is the lowest concentration of an antimicrobial at which a microorganism is completely killed. In this study, the MBC of zinc sulfate was 128 µg/ml for 42 isolates and 256 µg/ml (one-fold higher than the MIC) for the remaining 8 tested isolates.

### Time kill-kinetics assay

**Fig. 1:**
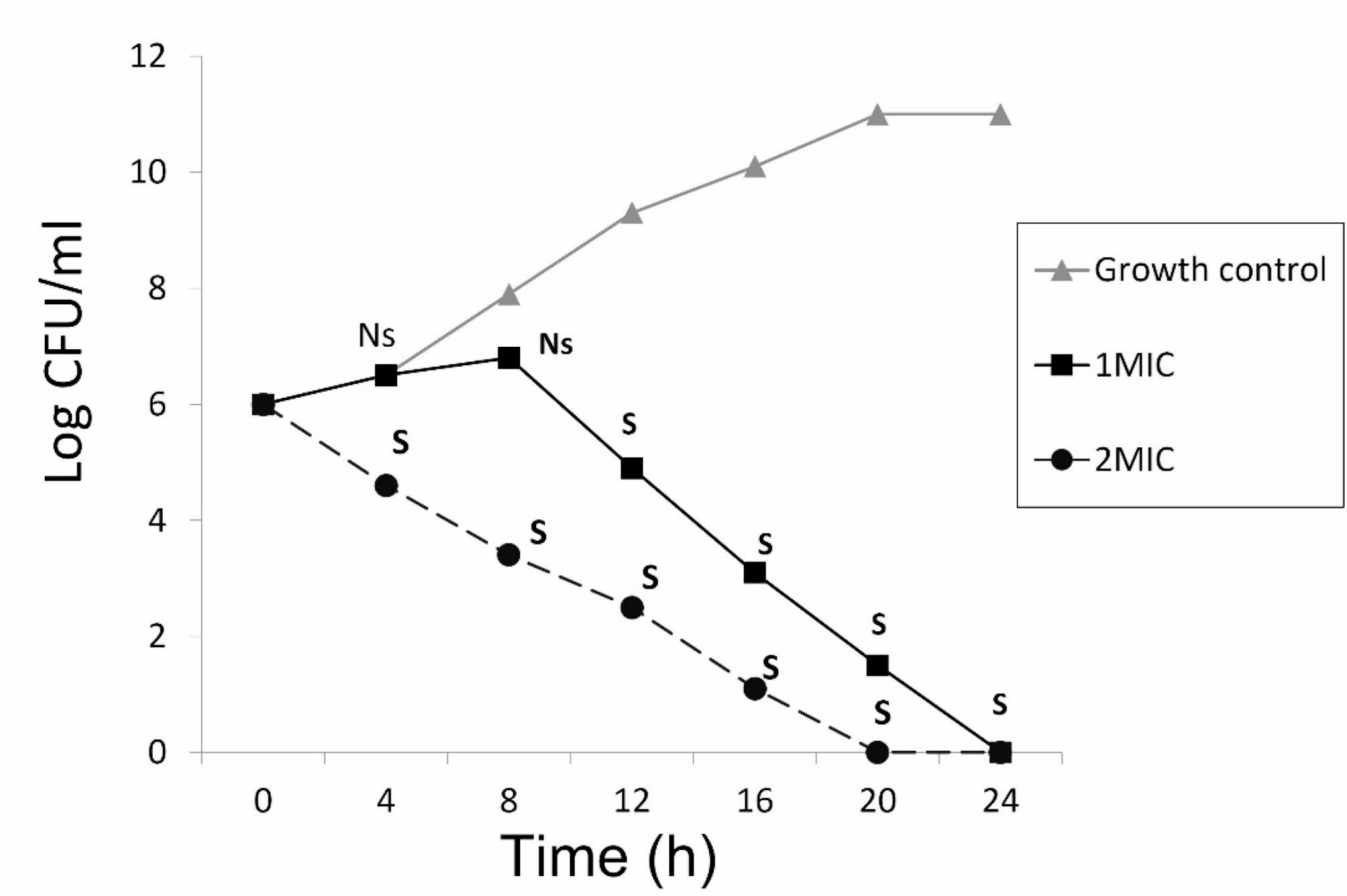
Time kill kinetics of zinc sulfate at MIC and 2×MIC against *S. aureus* isolates: S; means significant p-value (˂0.05) in comparison to growth control. NS; means non-significant p value (˃0.05) in comparison to growth control.

The time-kill kinetics study of the MIC and 2×MIC of zinc sulfate against the tested isolates showed a significant (P value ˂0.05) reduction in the number of CFU/ml over time, relative to the starting inoculum. Moreover, the MIC and 2×MIC of zinc sulfate exhibited bactericidal activity after the first 20 and 12 h, respectively as shown in Fig. 1.

### Antimicrobial susceptibility pattern of *S. aureus* isolates to different antibiotics without and in combination with zinc sulfate

**Fig. 2:**
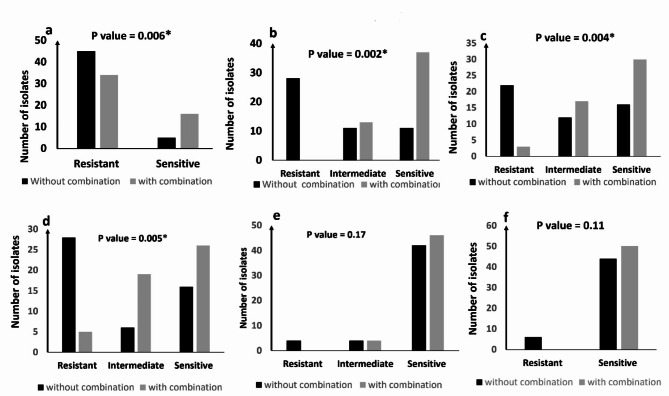
Antimicrobial susceptibility pattern of *S. aureus* isolates to different antibiotics without and in combination with zinc sulfate: a; Oxacillin, b; ceftaroline, c; vancomycin, d; azithromycin, e; teicoplanin, and f; linezolid. * Means significant p-value ˂0.05.

The MICs of the different antibiotics were determined by the broth microdilution method and the isolates were categorized as sensitive, intermediate, and resistant according to CLSI guidelines. Out of 50 *S. aureus* isolates tested, 28 (56%) were defined as MDR to the tested antibiotics. The distribution of *S. aureus* clinical isolates according to their MIC to oxacillin, ceftaroline, vancomycin, azithromycin, teicoplanin, and linezolid without and in combination with zinc sulfate showed that the MIC of different antibiotics against *S. aureus* clinical isolates decreased after combination with zinc sulfate and the MDR isolates decreased from 28 (56%) to 3(6%). The antimicrobial susceptibility of these isolates was significantly (P values ˂0.05) increased to the following antibiotics: oxacillin, ceftaroline, vancomycin, and azithromycin after combination with zinc sulfate as shown in Fig. 2. Also, the MIC of different antibiotics against *S. aureus* ATCC 29,213 decreased after combination with zinc sulfate from: 0.48, 0.24, 1, 2, 0.5 and 1 to 0.24, 0.12, 0.25, 0.5, 0.5 and 0.5 for oxacillin, ceftaroline, vancomycin, azithromycin, teicoplanin, and linezolid, respectively.

### The synergistic potential of zinc sulfate with the tested antibiotics

**Fig. 3:**
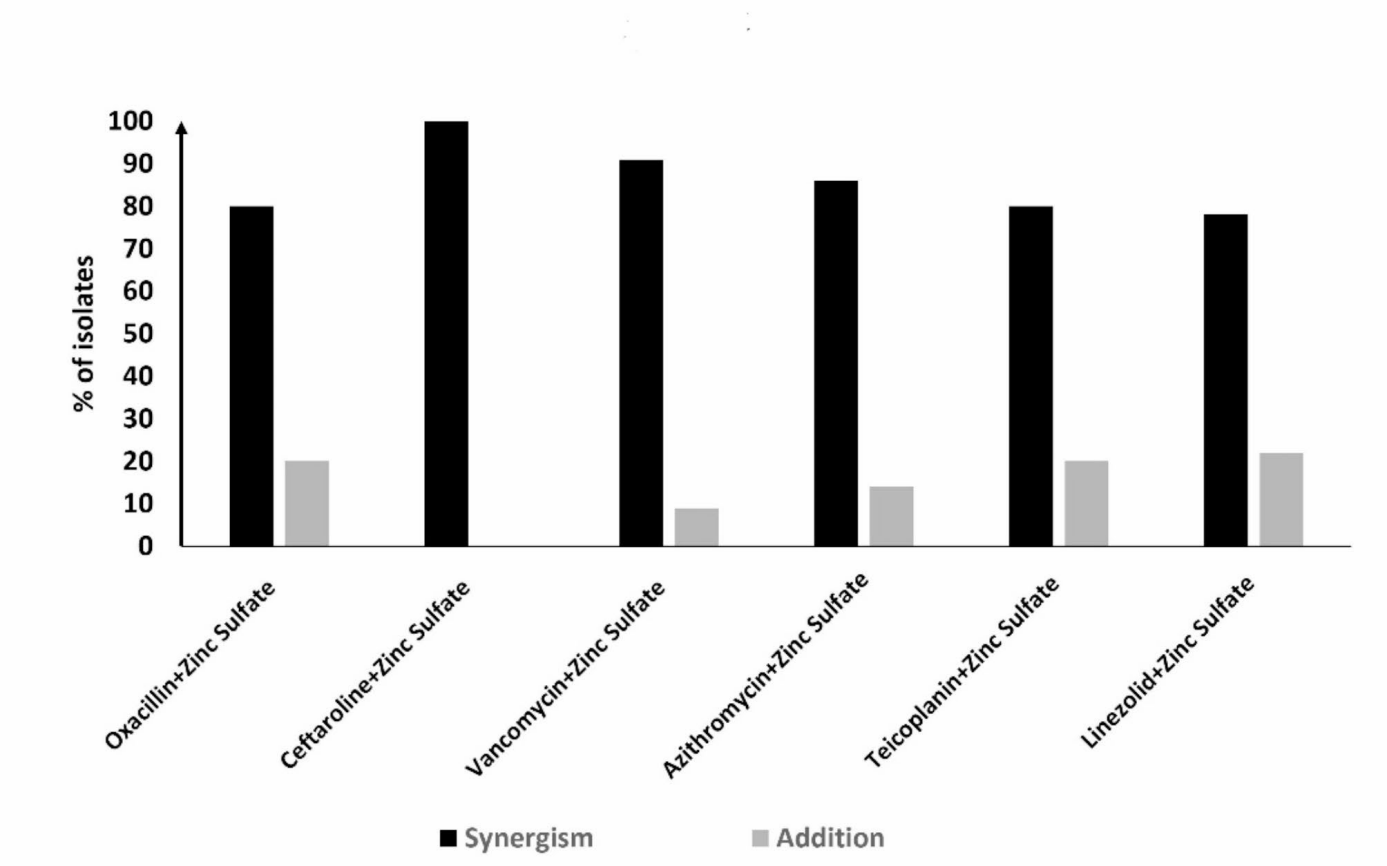
Results of the combinations of zinc sulfate and antibiotics. Interpretation of the checkerboard method reported either synergistic or additive effect, no indifference or antagonistic effect was detected at all.

The combination of zinc sulfate and antibiotics was tested by checkerboard microdilution assay. Synergy, addition, indifference, or antagonism were evaluated by determining the FICI. Importantly, zinc sulfate gave a synergistic effect when combined with the tested antibiotics as the mean FICI values were ≤ 0.5 for all tested antibiotics indicating a synergistic effect (Table 1). The best synergistic effect was reported with ceftaroline and zinc sulfate as 100% of the isolates show synergistic effects. An additive effect was reported with other antibiotics combined with zinc sulfate on a few numbers of isolates as shown in Fig. 3, but there is no indifference or antagonistic effect at all.

**Table 1:** The fractional inhibitory concentration (FIC) index of the tested antibiotic with zinc sulfate combination.

Compound	FICA Mean (Range)	FICB Mean (Range)	FICI Mean (Range)	Result
Oxacillin + Zinc Sulfate	0.18 (0.06–0.50)	0.19 (0.03–0.50)	0.37 (0.28-1.00)	Synergism
Ceftaroline _+_ Zinc Sulfate	0.15 (0.03–0.25)	0.13 (0.03–0.25)	0.28 (0.06–0.50)	Synergism
Vancomycin + Zinc Sulfate	0.17 (0.03–0.50)	0.16 (0.03–0.25)	0.33 (0.09–0.63)	Synergism
Azithromycin + Zinc Sulfate	0.14 (0.03–0.25)	0.16 (0.06–0.5)	0.30 (0.16–0.75)	Synergism
Teicoplanin + Zinc Sulfate	0.29 (0.16 -1.00)	0.12 (0.03–0.26)	0.41 (0.19–1.1)	Synergism
Linezolid +Zinc Sulfate	0.26 (0.13-1.00)	0.11 (0.03–0.26)	0.37 (0.16–1.03)	Synergism

Synergy was defined when the mean FICI was ≤ 0.5.

### Characterization of antibiofilm activity of zinc sulfate

**Fig. 4:**
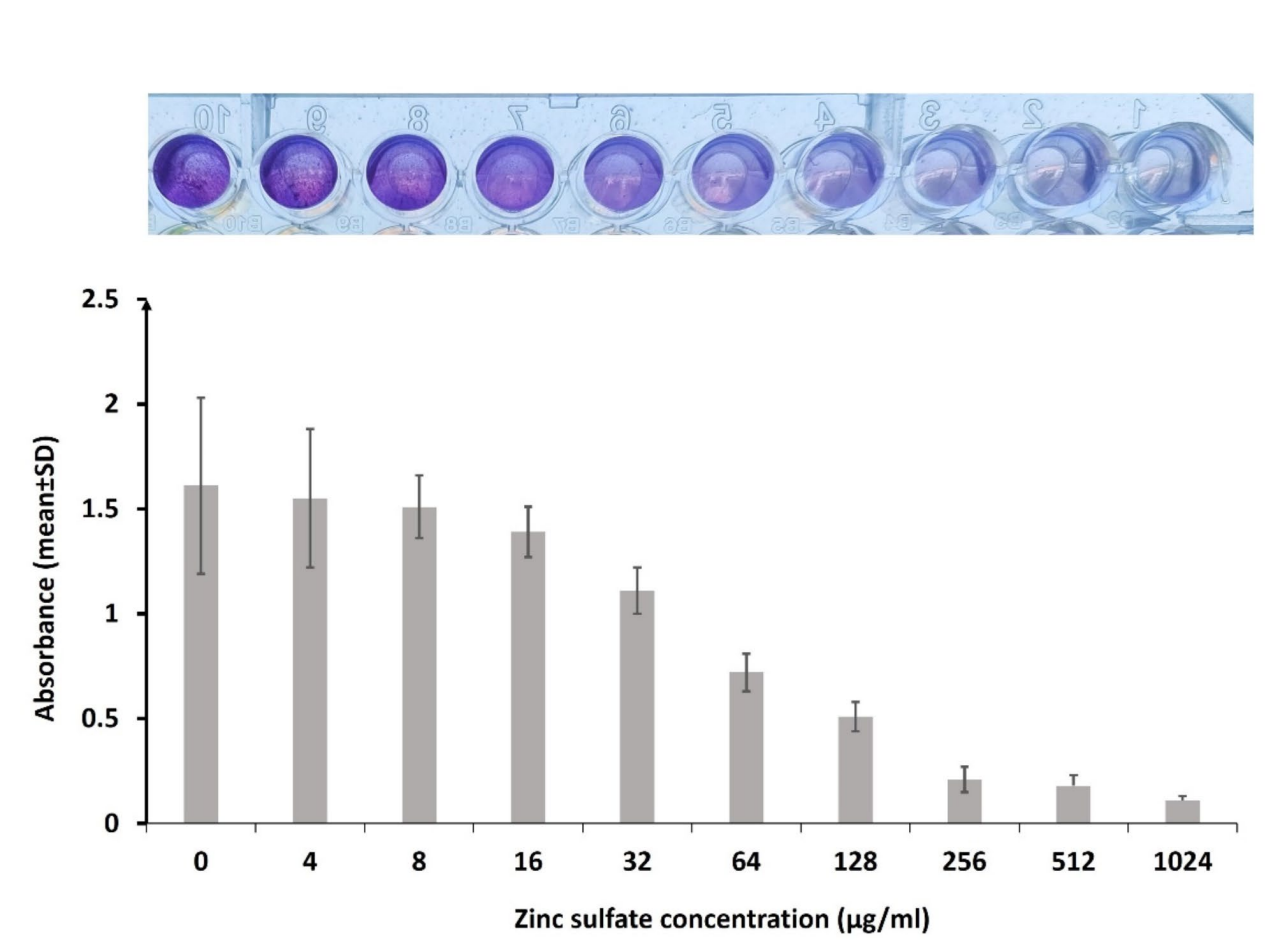
Effects of zinc sulfate on *S. aureus* biofilm formation: The antibiofilm effect of zinc sulfate against *S. aureus* isolates with and without zinc sulfate was characterized by crystal violet assay. S; means significant p value (˂0.05) in comparison to zero zinc sulfate concentration. NS; means non-significant p value (˃0.05) in comparison to zero zinc sulfate concentration. Images of crystal violet-stained biofilms in microtiter plate wells are shown.

The results of the microtiter plate method performed to assess biofilm formation of our isolates reported that 34 (68%) isolates were biofilm producers. Treating these isolates with zinc sulfate at a concentration of ≥ 32 µg/ml showed a significant reduction of the mean absorbance values, which means a decrease in the biofilm mass as shown in Fig. 4. Zinc sulfate significantly inhibited the biofilm production of *S. aureus* in a concentration-dependent manner. Zinc sulfate at ≥ 256 µg/ml concentration inhibited biofilm formation for all isolates. The absorbance of the wells and the concentration of zinc sulfate needed for inhibition of biofilm formation for each isolate were presented in supplementary table S1.

### Influence of zinc sulfate on the expression of *S. aureus* biofilm encoding genes without and with treating *S. aureus* isolates with sub-inhibitory concentration of zinc sulfate

The expression levels of biofilm genes; *ica A*,* D*,* B*,* and fnb A* were determined in both zinc sulfate-treated and untreated *S. aureus* clinical isolates by qRT-PCR, and the fold change was calculated. The expression of biofilm encoding genes represented by the mean fold change was significantly repressed in zinc sulfate-treated bacteria as compared to untreated cells (P value ˂ 0.05) as listed in Table 2.

**Table 2:** Biofilm encoding genes expression before and after treatment with zinc sulfate.

Gene expression	Before % (Number of isolates)	After % (Number of isolates)	FC before Mean (Range)	FC after Mean (Range)	*P* value
*IcaA*	100% (34/34)	91% (31/34)	132 (17:264)	18 (1:85)	0.007
*IcaD*	82% (28/34)	71% (24/34)	87 (5:201)	11 (2:25)	0.009
*IcaB*	79% (27/34)	76% (26/34)	35 (4:99)	6 (1:18)	0.010
*FnbA*	100% (34/34)	82% (28/34)	209 (29 :675)	34 (11:92)	0.002

% and number of isolates showed gene expression. FC, fold change before or after treating biofilm producing *S. aureus* isolates with zinc sulfate.

### Inhibition of *S. aureus* virulence factors by zinc sulfate

#### Hemolytic activity

**Fig. 5:**
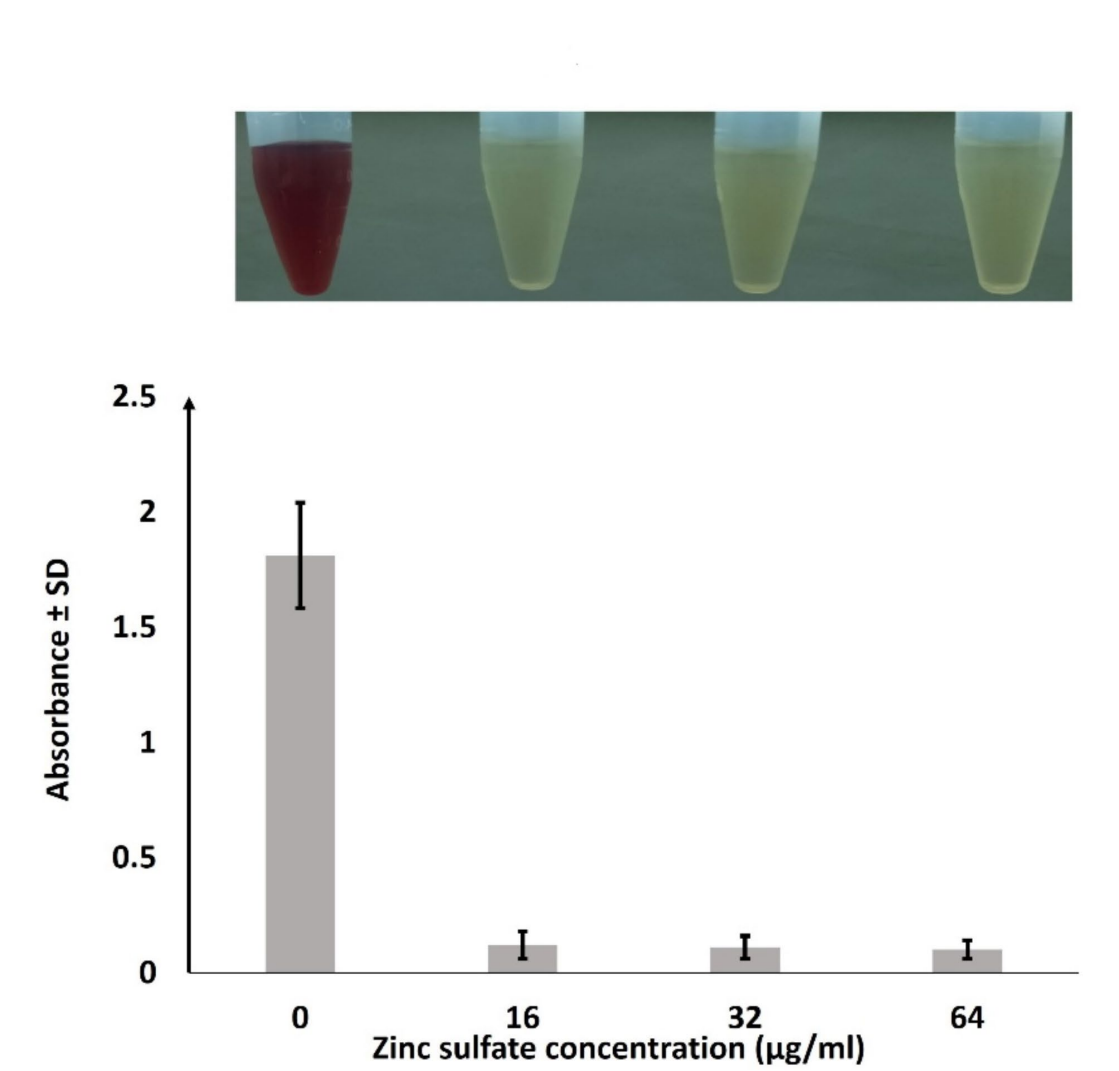
Effects of zinc sulfate on blood hemolysis by *S. aureus*. Hemolysis assays by *S. aureus* were performed using human red blood cells grown with or without zinc sulfate. S; means significant p value (˂0.05) in comparison to zero zinc sulfate concentration. Images of hemolytic activities in Eppendorf tubes are shown.

*S. aureus*-induced RBCs lysis was evaluated after incubating *S. aureus* cultures with the sub-MICs of zinc sulfate. Interestingly, zinc sulfate at all tested concentrations significantly inhibited the hemolysis of human red blood cells by all *S. aureus* isolates including *S. aureus* ATCC 29213, even at a low 16 µg/ml concentration, as shown in Fig. 5 and supplementary table S1. These findings indicate that prior treatment of *S. aureus* by zinc sulfate interferes with the hemolytic capability of *S. aureus*.

#### Coagulase activity

The coagulase activity of *S. aureus* was significantly (p-value ˂0.05) inhibited by zinc sulfate based on the increased time required for human plasma coagulation as shown in Table 3 and supplementary table S1. Zinc sulfate at 16 and 32 µg/ml produced an approximately 155- and 296- minutes lag in the coagulation time of human plasma produced by *S. aureus* coagulase enzyme, respectively, whereas 64 µg/ml zinc sulfate prevented coagulation until 10 h incubation. Coagulase enzyme activity by *S. aureus* ATCC 29213 was inhibited at all tested concentrations until 10 h incubation.

**Table 3:** Time required for human plasma coagulation by *S. aureus* with different zinc sulfate concentrations.

Zinc sulfate concentration	Time of coagulation in minutes (Mean ± SD)	*P* value
0	183 ± 23	-
16	338 ± 16	0.009*
32	479 ± 11	0.001*
64	˃600	˂0.001*

Results of the tube coagulation test for *S. aureus* isolates with and without zinc sulfate, * means significant p value ˂0.05 in comparison to zero zinc sulfate concentration.

#### Catalase activity

**Fig. 6:**
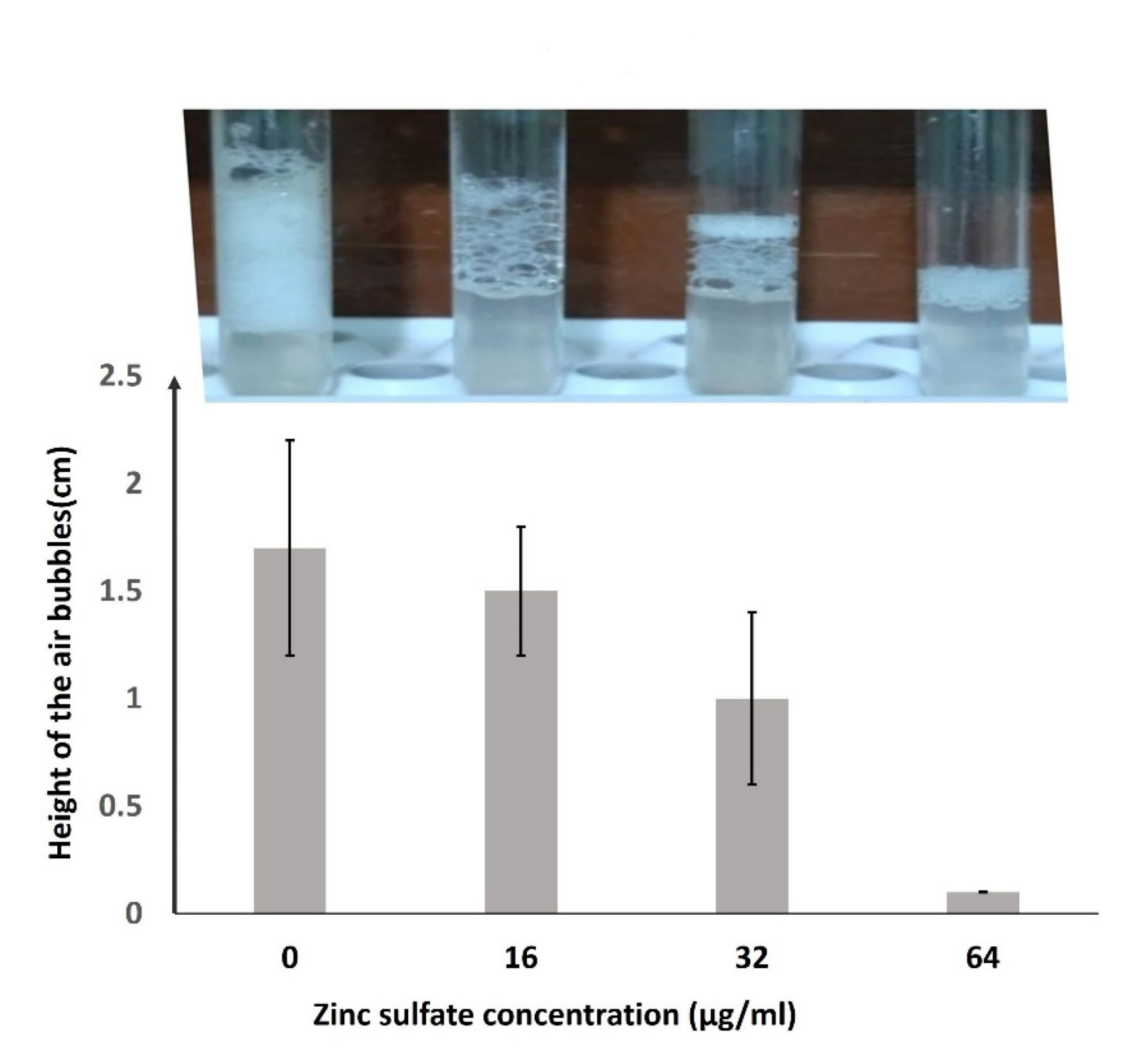
Effects of zinc sulfate on *S. aureus* catalase activity: For catalase assay, H_2_O_2_ was dispensed into the previously adjusted *S. aureus* cultures with and without zinc sulfate. The height of air bubbles released above the culture was measured in centimeters using a ruler. S; means significant p value (˂0.05) in comparison to zero zinc sulfate concentration. NS; means non-significant p value (˃0.05) in comparison to zero zinc sulfate concentration. Images of air bubble columns in glass tubes are shown.

Importantly, zinc sulfate exhibited a strong reduction in the catalase activity at 64 µg/ml which was noticed by a significant decrease in the mean height of the air bubbles produced by *S. aureus* cultures in the catalase assay as shown in Fig. 6 and supplementary table S1. Catalase enzyme activity by *S. aureus* ATCC 29,213 was significantly decreased after the application of zinc sulfate as the height of the air bubbles was reduced from 1.4 cm to 0.8, 0.5, 0.2 cm at 16, 32, and 64 µg/ml concentrations, respectively in the catalase assay.

## Discussion

*S. aureus* is a major bacterial human pathogen that causes numerous acute and chronic infections, ranging from minor skin infections to septicemia^1^. The present study included 50 isolates of *S. aureus*. The isolates were obtained from patients with surgical or burn-infected wounds. Out of 112 wound swabs, 50 swabs were identified as *S. aureus* isolates which represents 44.6%. This percentage is near that detected by other studies^7–9^. In this study, *S. aureus* isolates showed a higher resistance rate to oxacillin (90%), ceftaroline (56%), azithromycin (56%), and vancomycin (44%). Several similar studies done in Egypt reported a relatively similar antibiotic resistance rate to oxacillin^9–11^, but a relatively lower resistance rate to oxacillin (76%) was reported by Saeed et al.^12^. Abdelraheem et al. in Egypt reported 33.6 resistance rate to vancomycin which is comparable with the prevalence rate reported in the current study^10^. However, lower resistance rates of 20.7 and 14.8% were reported by Ahmed et al. and Ghoniem et al. in Egypt^11,13^. These discrepancies could be related to different geographical settings, different antibiotic prescription guidelines, and different sample sizes of these studies. In this study lower resistance rates were reported with teicoplanin (8%) and linezolid (12%), this result was relatively comparable to previous reports from Egypt which reported an 18.6% resistance rate to linezolid ^11^ and 13.9% resistance rate to teicoplanin^9^. The MDR phenotype was 56% in this study, which was close to the MDR rates (56% and 64%, respectively) reported in two studies conducted in Egypt^11,14^. *S. aureus* is a strong biofilm producer. The biofilm makes antimicrobial cannot reach *S. aureus*; it was noticed that the microorganisms that establish biofilm were 1000 times more resistant to antimicrobial agents compared to planktonic growth conditions^15^. In this study, the percentage of biofilm-producing *S. aureus* isolates was found to be 68%. The high percentage of biofilm-producing isolates reported in this study was consistent with other previous studies^9,10,16^. In these previous studies, the authors reported the percentage of biofilm producers among *S. aureus* isolates ranges between 70 and 80% of their isolates. Karki and Bimanand et al. reported a higher prevalence rate of *S. aureus* biofilm producers of 86.3 and 96%, respectively^17,18^.

With the emergence and spread of resistance to different antimicrobials, the development of novel and effective alternatives to traditional antibiotics has become an urgent need. Trying a safer alternative for antibiotics, particularly those that are natural and non-toxic is our goal in this study. Zinc is one such option that has no side effects, is inexpensive and is easily accessible. Zinc is one of the essential minerals that play an important role in different biological activities in humans. In this study, we have evaluated the antimicrobial effect of zinc sulfate by the microdilution method which reported that zinc sulfate has a good antibacterial effect against *S. aureus* clinical isolate at MIC of 128 µg/ml. The antimicrobial effect of zinc sulfate in the present investigation is in line with the findings of previous studies which reported that zinc sulfate exhibited a relatively increased antibacterial activity against *S. aureus* isolate with increasing its concentration^19,20^. In the previously mentioned studies, the authors have tested the antibacterial effect of zinc sulfate against *S. aureus isolates* by the well diffusion method, the diameter of the zone of inhibition around zinc sulfate increased with increasing its concentration. Also, our results reported that zinc sulfate gives a synergistic effect with all tested antibiotics against *S. aureus* isolates, hence it can be used as an antibiotic modifier to decrease the dose of antibiotics and to decrease the antibiotic resistance of this bacteria.

*S. aureus* is considered as an important human pathogen due to multiple virulence factors, in this study we have investigated the effect of zinc sulfate on *S. aureus* virulence factors. One of these virulence factors in *S. aureus* is biofilm formation, herein we studied the effect of zinc sulfate on 34 biofilm-producing S. *aureus* isolates. Zinc sulfate exhibited good anti-biofilm activity against *S. aureus* as it could inhibit biofilm formation for all isolates at 256 µg/ml concentration. The gene expression profiles of zinc sulfate-treated *S. aureus* were studied to further investigate the inhibitory effect of zinc sulfate on bacterial biofilm formation. Interestingly, treatment of *S. aureus* with zinc sulfate affects the expression of genes involved in biofilm formation. Biofilm-related genes (*icaADB and fnbA*) were significantly down-regulated in zinc sulfate-treated bacteria as compared to untreated cells. Therefore, the antibiofilm activity of zinc sulfate can be explained by interference with bacterial biofilm integrity or repression of biofilm encoding genes. This study is the first to examine the inhibitory effects of zinc sulfate on biofilm formation and virulence factor production in *S. aureus*. *S. aureus* produces abundant extracellular virulence factors and enzymes to invade and establish infections in the host system. The α-hemolysin is one such kind of exotoxin which is highly potent in the lysis of various cells such as. erythrocytes, monocytes, endothelial cells, and epithelial cells^21^. Importantly, *S. aureus*-induced lysis of RBCs was completely inhibited upon treatment of bacterial cells with zinc sulfate, zinc may bind to the enzymes and proteins responsible for hemolysis making them less accessible for binding to RBCs. To evaluate the anti-virulence approach of zinc sulfate, we have also investigated its effect on the coagulase and catalase activity of *S. aureus.* The coagulase activity of *Staphylococcus aureus* was inhibited by zinc sulfate based on the increased time required for plasma coagulation in a concentration-dependent way. Also, zinc sulfate in a dose-dependent manner inhibited the activity of the catalase enzyme produced by *S. aureus* which was noticed by a significant decrease in the height of the air bubbles in the catalase assay. The antibacterial and anti-virulence effect of zinc sulfate could be explained by previous studies that reported, zinc ion has several effects on bacteria and can inhibit numerous enzymes in bacterial cells^22,23^. Zinc ion capable of suppressing bacterial cell’s activities such as glycolysis, polysaccharide synthesis, transmembrane proton translocation, and acid tolerance^23^. It can reduce Adenosine Triphosphate (ATP) synthesis in bacterial cells due to its ability to inhibit the glycolytic enzymes; glyceraldehyde-3-phosphate dehydrogenases and pyruvate kinase as well as the phosphoenolpyruvate^24^. In conclusion, present data support the efficiency of zinc sulfate as an anti-bacterial, anti-virulence, and anti-biofilm agent in the treatment of *S. aureus* infections and possibly other important pathogens. This study recommends the incorporation of zinc sulfate as an adjuvant with other antibiotics targeting *S. aureus* based on the promising findings reported herein to control infection with this pathogen. We recommended further in vivo studies to explore the possible side effects of zinc sulfate. As well as a large molecular investigation on many *S. aureus* isolates is recommended to study the effect of zinc sulfate on virulence factors and antibiotic resistance gene expression.

## Methods

In this cross-sectional study, 50 non-repeated clinical isolates of *S. aureus* were isolated from patients with burn or surgical infected wounds at the plastic surgery department, of Minia University Hospital. All collected wound samples (*N* = 112) were cultured on nutrient agar, blood agar, and mannitol salt agar. Identification of *S. aureus* isolates was performed by colony morphology, Gram staining, and biochemical tests. Standard biochemical tests used for identification of *S. aureus* were catalase, coagulase, methyl red, and citrate positive; fermentation of glucose, lactose, and mannitol; Indole and VP test negative^25^. *S. aureus* isolates were confirmed by the identification of the *16s RNA* gene. *S. aureus* ATCC 2921 was also involved in this study as a reference strain.

### Determination of the antibacterial activity of zinc sulfate

The MIC of zinc sulfate against *S. aureus* was determined by using the broth microdilution method in 96-well microtiter plates according to CLSI guidelines. Zinc sulfate powder was purchased from Epico-Pharma company, Egypt. Zinc sulfate was suspended in sterile distilled water and constantly stirred until a uniform stock suspension was formed at a concentration of 2048 µg/ml. Serial dilutions were prepared in 10 wells, then 10 µl of bacterial suspension (adjusted to 0.5 MacFarland standard then diluted according to CLSI guidelines) were added to wells containing different concentrations of zinc sulfate, and the plates were then incubated overnight at 37 °C. Visible growth turbidity was then observed as compared with both a positive control well (well number 11 contains bacterial culture only and appears turbid) and a negative control well (well number 12 contains culture broth without bacteria and appears clear). The minimal inhibitory concentration (MIC) was defined as the lowest concentration of zinc sulfate that inhibits bacterial growth with no visible growth turbidity). After that, the minimum bactericidal concentration (MBC) was determined as follows: A sterile loop was used to subculture the nutrient agar plates from the wells of MIC up to the wells of the highest concentration of zinc sulfate. The concentration of zinc sulfate where no growth was detected is considered MBC for the bacterial strain tested.

### Determination of MIC of the tested antibiotics

Antimicrobial susceptibility testing to the following antibiotics oxacillin, ceftaroline, vancomycin, azithromycin, teicoplanin, and linezolid was done by microdilution method as mentioned before according to CLSI guidelines^26^. The isolates showing resistance to three or more of the tested antimicrobials were identified as multi-drug resistant (MDR) isolates.

### Time kill-kinetics study of zinc sulfate

Zinc sulfate was tested to detect the time-kill kinetics against *S. aureus* isolates over time according to CLSI guidelines. Briefly, a previously adjusted grown culture of Muller Hinton broth with the tested strains was inoculated with MIC and 2×MIC of zinc sulfate (starting inoculum was 1.0 × 10^6^ CFU/mL). Viable count on Muller Hinton agar plates was performed at each time point (0, 4, 8, 12, 16, 20 and 24 h) and incubated at 37 ºC. Growth control was performed for the tested strains without being treated by zinc sulfate. A graph of log_10_ CFU versus time was created. Bactericidal activity is defined as higher than 3 log_10_ fold colony forming unit decrease of the starting inoculum over time (killing of 99.9% of the starting inoculum)^27^.

### Checkerboard microdilution assay

A checkerboard assay was performed between zinc sulfate and different antibiotics against *S. aureus* isolates (*N* = 50). The assay is used to determine the impact on potency of the combination of zinc sulfate and antibiotics in comparison to their individual activities. Briefly, in a microtiter plate, columns 1 to 11 contain 2-fold serial dilutions of the tested antibiotic and rows A to G contain 2-fold serial dilutions of zinc sulfate. Column 12 contains a serial dilution of zinc sulfate alone, while row H contains a serial dilution of antibiotic alone. Ten microliters of bacterial suspension (adjusted to 0.5 MacFarland standard) were added to wells. The Fractional Inhibitory Concentration (FIC) index value was used to interpret the interactions between the antibiotics being tested and zinc sulfate according to the following equation ^28^:

FIC Index = FICA + FICB.

$$\:\text{F}\text{I}\text{C}\text{A}\:\left(\text{o}\text{f}\:\text{a}\text{n}\text{t}\text{i}\text{b}\text{i}\text{o}\text{t}\text{i}\text{c}\right)\:=\:\frac{\text{M}\text{I}\text{C}\:\text{o}\text{f}\:\text{a}\text{n}\text{t}\text{i}\text{b}\text{i}\text{o}\text{t}\text{i}\text{c}\:\text{i}\text{n}\:\text{c}\text{o}\text{m}\text{b}\text{i}\text{n}\text{a}\text{t}\text{i}\text{o}\text{n}\:}{\text{M}\text{I}\text{C}\:\text{a}\text{n}\text{t}\text{i}\text{b}\text{i}\text{o}\text{t}\text{i}\text{c}\:\text{a}\text{l}\text{o}\text{n}\text{e}}$$

$$\:\text{F}\text{I}\text{C}\text{B}\:\left(\text{o}\text{f}\:\text{z}\text{i}\text{n}\text{c}\:\text{s}\text{u}\text{l}\text{f}\text{a}\text{t}\text{e}\right)\:=\:\frac{\text{M}\text{I}\text{C}\:\text{o}\text{f}\:\text{z}\text{i}\text{n}\text{c}\:\text{s}\text{u}\text{l}\text{f}\text{a}\text{t}\text{e}\:\text{i}\text{n}\:\text{c}\text{o}\text{m}\text{b}\text{i}\text{n}\text{a}\text{t}\text{i}\text{o}\text{n}\:}{\text{M}\text{I}\text{C}\:\text{z}\text{i}\text{n}\text{c}\:\text{s}\text{u}\text{l}\text{f}\text{a}\text{t}\text{e}\:\text{a}\text{l}\text{o}\text{n}\text{e}}$$

The FIC Index value is then used to categorize the interaction of the two antimicrobials tested as follows: Synergy was defined when FICI was ≤ 0.5, additive in which 0.5˂ FICI ≤ 1, indifferent in which FICI is ˃1 ˂4, whereas antagonism when the FICI is ˃4 ^28^.

### Assessment of *S. aureus* biofilm formation by the microtiter plate method

The ability of *S. aureus* clinical isolates to form biofilm was tested according to Stepanović et al.^29^ with some modifications. *S. aureus* overnight cultures were prepared in Trypticase soy broth supplemented with 1% glucose and then adjusted to 0.5 MacFarland standard. A suspension of 200 µL/well of the adjusted broth cultures was added to each well of 96 well microtiter plates and incubated for 48 h at 37 °C to allow for biofilm formation. Negative control wells that contained 200 µL/well sterile broth was included in each plate. The formed *S. aureus* biofilms were fixed using methanol for 20 min. After fixation, biofilms were stained with 1% crystal violet for 15 min. The plates were air-dried, and the bound dye was dissolved by 95% ethyl alcohol. The absorbance of the wells was measured by ELISA Reader (Bio Tek, USA) at 570 nm. The cut-off value was calculated as three times standard deviations above the mean absorbance of the negative control. The tested strains were defined as biofilm producers when the absorbance of the corresponding well was > cut-off value.

### Assay of the anti-biofilm effect of zinc sulfate by microtiter plate method

The antibiofilm potential of zinc sulfate against *S. aureus* clinical isolates was characterized as follows: overnight grown broth cultures of bacteria were diluted to 0.5 MacFarland standard. Then aliquots of about 100 µL were added to wells of sterile microtiter plates that contained different concentrations of zinc sulfate ranging from 1024 to 0 µg /ml. The plate was incubated at 37 °C for 48 h. The influence of zinc sulfate on bacterial biofilm formation was assessed as described above.

### Effect of zinc sulfate on biofilm genes expression

Gene expression of biofilm encoding genes (*ica A*,* ica B*,* ica D*, and *fnb A*) was assessed using quantitative real-time reverse transcriptase-polymerase chain reaction (rt-PCR) without and with treating *S. aureus* isolates with sub-inhibitory concentration of zinc sulfate (1/2 MIC). Two tubes of bacterial-grown nutrient broth cultures for each isolate were prepared and adjusted to 0.5 McFarland standards, and then sub-MIC of zinc sulfate was added to one of them. Then, all tubes were incubated for 24 h at 37 °C. After 24 h incubation, bacterial RNA was extracted from the prepared grown cultures for all isolates by total RNA extraction kit (Zymo Research CORP, Australia) according to the manufacturer’s instructions. One-step RT qPCR kits (Enzynomics, Korea) were used to amplify and quantify the extracted RNA of the target genes using the Real-Time PCR system (Applied Biosystem, USA) according to the manufacturer’s instructions. The expression level of each gene was normalized to the reference gene (16 S rRNA) and reference sample. The sequence of primers used in this study was selected from a previously published study^10^. The fold change of each target gene was calculated using the equation; 2 − ΔΔCt as described previously^30^.

### Evaluation of zinc sulfate inhibitory effect on *S. aureus* virulence factors

#### Hemolysis assay

Hemolysis was assayed as previously described with some modification*s*^31^. Briefly, fresh human blood was collected in an anticoagulant solution (EDTA) and spun at 3000 rpm for 10 min. Red blood cells were collected and plasma with a buffy coat was discarded. Separated red blood cells were washed with PBS buffer 3 times diluted in PBS buffer. The previously prepared different nutrient broth cultures of *S. aureus* (adjusted to 0.5 MacFarland standard) with different zinc sulfate concentrations (0, 16, 32, and 64 µg/ml) were incubated at 37^°^C for 16 h at 250 rpm. Bacterial cultures were added to the washed human red blood cells, mixed, and incubated at 37^°^C for 1 h at 250 rpm. Supernatants were collected by centrifugation and optical densities of the supernatants were measured at 540 nm.

#### Coagulation assay

To evaluate the effect of zinc sulfate on the coagulase activity of *S. aureus*, a tube coagulation test was done as follows: 100 µl human plasma, 100 µl *S. aureus* nutrient broth culture (adjusted to 0.5 MacFarland standard), and 100 µl of the tested zinc sulfate concentration (0, 16, 32, or 64 µg/ml) were mixed in a test tube. Then this mixture was incubated at 37^°^C and the samples were checked for coagulation every 15 min for 10 h.

#### Catalase activity assay

For zinc sulfate effect on catalase activity, *S. aureus* nutrient broth cultures (adjusted to 0.5 MacFarland standard) with and without sub-MIC of zinc sulfate (16, 32, or 64 µg/ml) were incubated overnight in a shaking incubator at 200 rpm. H_2_O_2_ (3%) was dispensed into one ml of the overnight culture broth in a glass tube and mixed immediately. The height of air bubbles released above the culture was measured in centimeters using a ruler.

#### Statistical analysis

All data collected in this study were stored in an Excel sheet. Statistical analysis was done on SPSS package version 23.0 (SPSS Inc., Chicago, IL, USA). Continuous data are expressed as the mean, standard deviation (SD) or range. Categorical data are presented in the form of numbers and percentages. Chi-squared tests were performed for comparison of categorical data. Paired sample T test or Mann-Whitney test were performed for comparison of continuous data. A two-tailed p-value of ˂ 0.05 was considered statistically significant.

## Electronic supplementary material

Below is the link to the electronic supplementary material.

Supplementary Material 1

## Data Availability

All data generated or analyzed during this study are included in this published article.
