# Supplementary material for: Evaluation of anti-biofilm and anti-virulence effect of zinc sulfate on Staphylococcus aureus isolates
Source: Sci Rep. 2024 Oct 28;14:25747. doi: 10.1038/s41598-024-75317-0 (PMC11519333; doi:10.1038/s41598-024-75317-0)
Supplement: Supplementary file 1 — Supplementary Material 1 [file 41598_2024_75317_MOESM1_ESM.pdf]

Evaluation of anti-biofilm and anti-virulence effect of Zinc sulfate on *Staphylococcus aureus* isolates

***Wedad M. Abdelraheem[1], Heba S. Kamel[2], Aya Nabil Gamil[1]**

[1]Medical Microbiology and Immunology department- Faculty of Medicine- Minia University, Minia, Egypt; [2]Department of Biochemistry, Faculty of Medicine, Minia University, Minia, Egypt.

Supplementary Table S1: Anti-biofilm and anti-virulence factors effect of zinc sulfate on all tested isolates

[1]Strain ID	[2]Biofilm absorbance	[3]Zn sulfate conc. Inhibit biofilm	[4]Hemolysis absorbance	[5]Zn sulfate conc. Inhibit hemolysis	[6]Coagulation time without Zn sulfate	[7]Coagulation time with Zn sulfate (ug/ml)			[8]Catalase without Zn sulfate	[9]Catalase with Zn sulfate (ug/ml)		
						16	32	64		16	32	64
1	2.1	256	1.9	All tested conc	210	345	495	>600	2.2	1.8	1.2	0.3
2	2	256	1.9	All tested conc	195	330	480	>600	2.3	1.8	1.1	0.2
3	2.1	256	2	All tested conc	210	345	495	>600	2.1	1.8	1.2	0.1
4	1.6	256	1.5	All tested conc	180	345	480	>600	1.9	1.6	1.2	0.1
5	0.9	128	1.6	All tested conc	210	345	495	>600	1.5	1.2	0.9	0.1
6	0.8	128	1.7	All tested conc	195	345	480	>600	1.7	1.2	0.9	0.1
7	0.4	Non-biofilm	1.7	All tested conc	150	330	480	>600	2.3	1.6	1.1	0.1
8	1.3	256	1.8	All tested conc	165	300	465	>600	2.2	1.5	1	0.1
9	1.4	265	1.9	All tested conc	195	360	495	>600	2.1	1.4	1	0.1
10	1.9	256	2.1	All tested conc	180	345	495	>600	1.6	1.1	0.6	0.0
11	2	265	2.1	All tested conc	210	360	495	>600	1.7	1.3	0.9	0.1
12	0.2	Non-biofilm	2	All tested conc	210	345	495	>600	1.2	0.8	0.5	0.0
13	0.3	Non-biofilm	1.8	All tested conc	195	360	480	>600	1.7	1.1	0.6	0.1
14	0.8	128	1.5	All tested conc	180	300	465	>600	1.9	1.2	0.7	0.0
15	2.3	256	1.7	All tested conc	180	345	480	>600	1.7	1.3	0.7	0.1
16	1.4	256	1.9	All tested conc	180	330	480	>600	1.5	1.1	0.6	0.0
17	1.9	256	1.5	All tested conc	195	330	495	>600	1.6	1	0.6	0.1
18	0.4	Non-biofilm	1.4	All tested conc	210	360	495	>600	1.8	1.3	0.8	0.0
19	0.3	Non-biofilm	2.1	All tested conc	195	300	465	>600	2.3	1.8	1.2	0.3
20	0.9	128	1.4	All tested conc	195	345	465	>600	2	1.7	1.2	0.3
21	0.3	Non-biofilm	1.5	All tested conc	165	330	465	>600	2.3	1.8	1.1	0.3
22	2.2	256	1.6	All tested conc	165	360	465	>600	2.1	1.8	1.2	0.3
23	2.1	256	1.8	All tested conc	135	330	465	>600	2.3	1.6	1.2	0.1
24	0.4	Non-biofilm	1.9	All tested conc	180	360	495	>600	2.3	1.7	1.4	0.1
25	1.9	256	1.4	All tested conc	195	360	495	>600	2.3	1.7	1.1	0.2
26	0.3	Non-biofilm	1.8	All tested conc	135	330	480	>600	2.1	1.5	1.2	0.2
27	0.2	Non-biofilm	1.9	All tested conc	210	360	495	>600	2.3	1.6	1.3	0.1

28	0.3	Non-biofilm	1.8	All tested conc	195	345	465	>600	2.3	1.7	1.3	0.1
29	1.8	256	1.7	All tested conc	135	345	465	>600	1.4	0.9	0.5	0.0
30	0.3	Non-biofilm	1.4	All tested conc	135	330	465	>600	1.2	0.8	0.5	0.0
31	0.3	Non-biofilm	1.7	All tested conc	150	330	465	>600	1.9	1.4	0.9	0.1
32	1.4	256	1.8	All tested conc	195	345	465	>600	1.2	0.8	0.5	0.1
33	0.5	Non-biofilm	2	All tested conc	195	345	465	>600	1.3	0.8	0.5	0.0
34	2.1	256	2.1	All tested conc	210	360	495	>600	1.9	1.5	0.9	0.0
35	1.2	256	1.9	All tested conc	150	330	480	>600	1.9	1.4	0.9	0.1
36	1.5	256	1.7	All tested conc	180	315	480	>600	1.4	1	0.8	0.1
37	0.3	Non-biofilm	2.1	All tested conc	195	330	480	>600	1.6	1	0.6	0.1
38	2.2	256	2.1	All tested conc	180	315	465	>600	1.8	1.3	1	0.2
39	2	256	1.6	All tested conc	135	315	465	>600	2	1.7	1.6	0.2
40	1.6	256	1.5	All tested conc	150	300	465	>600	1.4	0.9	0.5	0.1
41	0.3	Non-biofilm	2	All tested conc	180	330	480	>600	1.6	1.1	0.9	0.2
42	1.9	256	2	All tested conc	180	360	495	>600	1.2	1	0.6	0.0
43	0.9	128	2.1	All tested conc	210	360	495	>600	1.7	1.3	0.9	0.1
44	1.6	265	1.7	All tested conc	195	345	480	>600	1.6	1.1	0.6	0.0
45	1.3	256	1.6	All tested conc	195	345	495	>600	1.9	1.4	0.9	0.1
46	1.8	256	1.9	All tested conc	195	345	495	>600	2.3	1.7	1.2	0.2
47	1.6	256	1.7	All tested conc	210	360	495	>600	1.5	1	0.5	0.0
48	0.3	Non-biofilm	2.1	All tested conc	195	360	480	>600	2.1	1.7	1.3	0.3
49	1.9	256	1.5	All tested conc	180	345	495	>600	2.2	1.9	1.6	0.3
50	2.1	256	1.6	All tested conc	180	300	465	>600	1.7	1.2	0.9	0.0

[1] strain ID number. [2] The absorbance of crystal violet stained well of the corresponding strain at 570 nm of the biofilm assay, [3] Minimal Zinc (Zn) sulfate concentration Inhibit biofilm, so the absorbance decreases to ≤0.6. [4] Absorbance of the supernatants of the hemolysis assay for the corresponding strain at 540. [5] Zn sulfate concentration inhibits hemolysis, so the supernatant appears yellow, and the absorbance decreases below 0.5 at 540 nm. [6]Coagulation time in minutes of the corresponding strain tested without Zn sulfate. [7] Coagulation time in minutes of the corresponding strain after being treated with different Zn sulfate concentrations (ug/ml). [8] The height of the air bubbles measured in cm in the catalase assay of the corresponding strain being without treated Zn sulfate. [9] The height of the air bubbles measured in cm in the catalase assay of the corresponding strain after being treated with different Zn sulfate concentrations (ug/ml).
